# Partial Ventilatory Support Modalities in Acute Lung Injury and Acute Respiratory Distress Syndrome—A Systematic Review

**DOI:** 10.1371/journal.pone.0040190

**Published:** 2012-08-16

**Authors:** Sarah M. McMullen, Maureen Meade, Louise Rose, Karen Burns, Sangeeta Mehta, Robert Doyle, Dietrich Henzler, for the Canadian Critical Care Trials Group (CCCTG)

**Affiliations:** 1 Department of Anesthesiology and Critical Care Medicine, Dalhousie University, Halifax, Canada; 2 Departments of Medicine and Clinical Epidemiology & Biostatistics, McMaster University, Hamilton, Canada; 3 Lawrence S. Bloomberg Faculty of Nursing, University of Toronto, Toronto, Canada; 4 Interdepartmental Division of Critical Care, University of Toronto and St Michael's Hospital, and Li Ka Shing Knowledge Institute, Toronto, Canada; 5 Department of Medicine and Interdepartmental Division of Critical Care Medicine, Mount Sinai Hospital, University of Toronto, Toronto, Canada; University of Tübingen, Germany

## Abstract

**Purpose:**

The efficacy of partial ventilatory support modes that allow spontaneous breathing in patients with acute lung injury (ALI) and acute respiratory distress syndrome (ARDS) is unclear. The objective of this scoping review was to assess the effects of partial ventilatory support on mortality, duration of mechanical ventilation, and both hospital and intensive care unit (ICU) lengths of stay (LOS) for patients with ALI and ARDS; the secondary objective was to describe physiologic effects on hemodynamics, respiratory system and other organ function.

**Methods:**

MEDLINE (1966–2009), Cochrane, and EmBase (1980–2009) databases were searched using common ventilator modes as keywords and reference lists from retrieved manuscripts hand searched for additional studies. Two researchers independently reviewed and graded the studies using a modified Oxford Centre for Evidence-Based Medicine grading system. Studies in adult ALI/ARDS patients were included for primary objectives and pre-clinical studies for supporting evidence.

**Results:**

Two randomized controlled trials (RCTs) were identified, in addition to six prospective cohort studies, one retrospective cohort study, one case control study, 41 clinical physiologic studies and 28 pre-clinical studies. No study was powered to assess mortality, one RCT showed shorter ICU length of stay, and the other demonstrated more ventilator free days. Beneficial effects of preserved spontaneous breathing were mainly physiological effects demonstrated as improvement of gas exchange, hemodynamics and non-pulmonary organ perfusion and function.

**Conclusions:**

The use of partial ventilatory support modalities is often feasible in patients with ALI/ARDS, and may be associated with short-term physiological benefits without appreciable impact on clinically important outcomes.

## Introduction

### Rationale

Mechanical ventilation restores gas exchange in patients with respiratory failure. In patients with acute lung injury (ALI) and acute respiratory distress syndrome (ARDS), controlled modes of mechanical ventilation that deliver full ventilatory support have traditionally been used to completely offload the work of breathing and thereby “rest the lung” and diaphragm [1]. During controlled ventilation, the ventilator performs the entire work of breathing and patients are generally sedated, and/or chemically paralyzed, prohibiting spontaneous breathing efforts. Recent trials of patients with ARDS suggest that upward of 30% of patients are paralyzed with neuromuscular blockers and managed with controlled modes of ventilation during the acute phase of respiratory failure [2], [3].

Partial ventilatory support, wherein the degree of mechanical support provided is adjusted to patient need, preserves diaphragmatic contraction and allows spontaneous breathing efforts. Traditionally reserved for use in weaning patients from mechanical ventilation [4], partial ventilatory support modes are now used in all phases of ventilation [5]. Accordingly, partial ventilatory support has been investigated in both animals and humans during the acute phases of lung injury, although uncertainty remains as to whether or not its use is beneficial or even feasible in patients with ALI and ARDS.

### Objectives

The primary objective of this scoping review was to systematically assess whether partial ventilatory support modalities can be used effectively in the early phase of ALI and ARDS [6], and whether or not their application in this patient population improves survival or reduces the duration of mechanical ventilation and lengths of stay in ICU and hospital. The secondary objective was to describe the physiologic effects with respect to hemodynamic stability and respiratory system function.

## Methods

### Search Strategy

The scope of studies included for review was deliberately broad. We searched for randomized controlled trials (RCTs), controlled observational studies (retrospective and prospective), case series and pre-clinical studies. Since there is no widely accepted definition of “partial ventilatory support”, we focused on modes allowing preserved spontaneous breathing activity during mechanical ventilation. In the setting of ALI/ARDS the following modes have been evaluated: assist-control (A/C), synchronized intermittent mandatory ventilation (SIMV), pressure support ventilation (PSV), proportional assist ventilation (PAV), airway pressure release ventilation (APRV), biphasic positive airway pressure (BIPAP) or neurally adjusted ventilatory assist (NAVA). APRV and BIPAP and their differences have been previously highlighted [7], [8], and henceforth will be referred to as APRV/BIPAP. MEDLINE, Cochrane, and EmBase databases (1966–February 2009) were searched using a predefined search strategy (see File S1); additionally, reference lists from relevant reviews, observational studies and clinical trials were hand searched.

### Data abstraction

Three authors (SMM, RD, DH) independently screened titles and abstracts, resolving disagreements by consensus. All studies judged by any reviewer as potentially relevant underwent full-text review to ascertain eligibility and were systematically categorized and analyzed by two reviewers (SMM, DH).

### Selection

Trials that evaluated human adults in the active treatment (i.e., acute, non-weaning) phase of respiratory failure from ALI and ARDS were included; pediatric and neonatal studies were excluded, as were those pertaining to ventilatory weaning, chronic ventilation, and non-invasive or extracorporeal modes of lung assist.

### Study characteristics

Primary outcomes of interest were ICU and hospital mortality, ICU and hospital and lengths of stay (LOS), duration of mechanical ventilation and/or ventilator-free days (VFDs). Secondary outcomes were hemodynamic variables (cardiac output, mean arterial pressure); impact on organ function (renal, gastrointestinal); gas exchange (including the ratio of partial pressure of oxygen [PaO_2_] to fractional inspired oxygen [FiO_2_] i.e., P/F ratio, and ventilation-perfusion [V/Q] matching); measures of respiratory mechanics (tidal volume, minute ventilation, airway pressures, work of breathing); effects on the development of ventilator associated lung injury (VALI); measures of patient comfort (as indicated by sleep hygiene and patient-ventilator synchrony, for example) and usage of analgesic and sedative medications.

### Validity assessment

We used a modified version of the Oxford Centre for Evidence-Based Medicine Levels of Evidence system (“the Oxford System”) [9] to evaluate studies of interest. A weakness of this system is the grading of systematic reviews (1a, 2a, 3a) that refers only to the source data (RCT, cohort, case series), but does not take into consideration the quality of the review, which may or may not have been systematic. Therefore, we modified the grading system [10] (Table 1), and introduced categories to represent physiologic studies in both humans (4b, i.e. those examining physiological impacts only without evaluations of outcomes) and animals [6]. These were rated as higher evidence grade than expert opinion because of their explicit foundation in scientific principles. While animal studies were included in the search strategy, we reviewed and considered these as supporting sources of data only.

**Table 1:** Modified Oxford Centre for Evidence-Based Medicine Levels of Evidence (8).

1a	Multicentre RCT/Meta-Analysis/SR of RCTs
	Clear search strategy, appraisal by ≥2 reviewers using published grading scheme for RCTs
1b	High quality, individual RCT
2a	SR of controlled cohort studies, or missing one criteria for SR in RCTs
2b	Prospective cohort/lower quality RCT
3a	SR of case control studies, or missing one criteria of SR in cohort study
3b	Retrospective cohort/case control study
4a	Case series/low quality cohort studies/low quality case control studies
4b	Physiological study (humans)
5	Pre-clinical study (animal)
6a	Review of the literature, *without* documented or systematic methodology
6b	Expert opinion/case report/technical note

RCT = randomized control trial; SR = systematic review. For definitions refer to text.

## Results

### Flow of included studies

**Figure 1:**
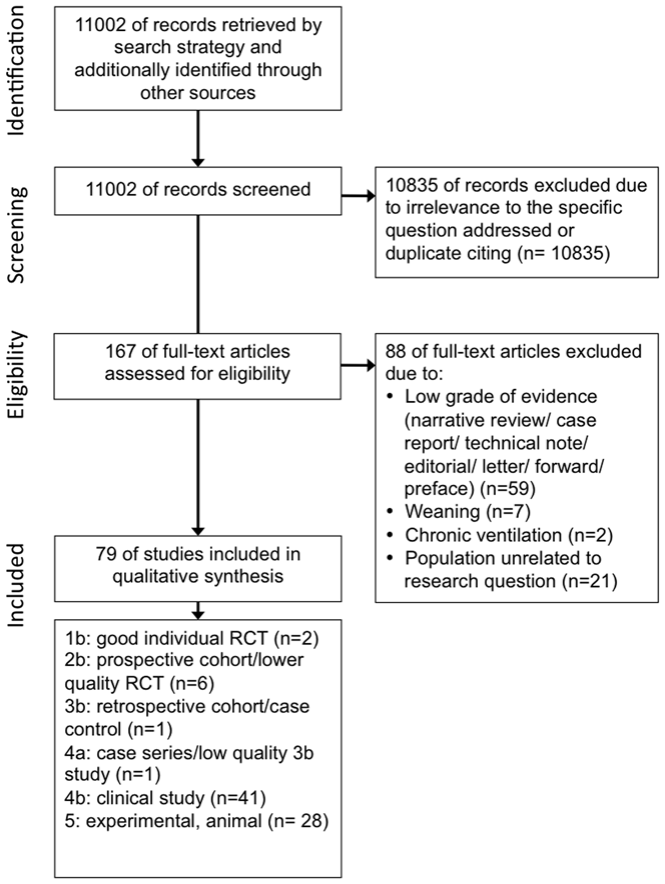
Flow of Included Studies.

The initial search yielded 11,002 articles, of which 10,835 were excluded through title and abstract review (Figure 1), leaving 167 articles potentially meeting our inclusion criteria. Of these, 88 were excluded because they were not relevant to the research question. The remaining 79 studies that were evaluated were: two RCTs (level 1b: [11], [12]), six prospective single-cohort studies (level 2b: [13]–[18]), one retrospective cohort study (level 3b: [19]), and one case series (level 4a: [20]). The remaining 69 studies included 41 physiologic studies in humans (level 4b) and 28 pre-clinical studies in animals (level 5). A summary of partial support modes investigated in the setting of ALI/ARDS is presented in Table 2.

**Table 2:** Summary of investigations by mode of ventilation.

	Gas exchange and ventilation	Hemodynamics	Respiratory mechanics, work of breathing	Patient-ventilator interaction, other effects
**AC** Assist Control	PC ± spontaneous breathing to assess intrapulmonary shunt fraction [54]; P-ACV vs. BIPAP vs. PSV [29]	P-ACV vs. BIPAP vs. PSV [28]; ARPV vs. PCV [12].	A/C vs. SIMV [43]; A/C vs. PAV [77]; A/C vs. PS, physiologic parameters and mechanoreceptor reflexes [80]	A/C vs. APRV/BIPAP, effects on analgo-sedative doses, sedation status [17] Pat.-ventilator interaction [38]
**SIMV** Synchronized Intermittent Mandatory Ventilation	SIMV vs. CMV [31]; SIMV vs. APRV [16], [11]		APRV vs. SIMV+PS, effects on respiratory system mechanics [11]	SIMV vs. PSV for changes in metabolic variables [73]; SIMV vs. adaptive support dys-synchrony [24]
**PSV** Pressure Support Ventilation	PSV vs. A/C vs. CMV [46]; PSV vs. APRV/BIPAP [59], [79] PSV vs. CMV [60], [51]; PSV vs. PAV (ventilation) [80] PSV vs. PAV (gas exchange) [81]; PSV vs. PSV+sighs vs. APRV/BIPAP vs. noisy PSV [54]; PSV vs. BIPAP/APRV vs. CMV [56]	PSV vs. A/C vs. CMV [46]; PSV vs. PAV [39]; PSV vs. CPAP vs. APRV/BIPAP for cardiac output, oxygen consumption [61], [58]	PSV vs. A/C vs. CMV [46]; PSV vs. CMV for changes in workloads, dynamic compliance [25]; determination of workload relief during PSV [82]; PSV vs. PAV for workload relief [70], [71]; PSV vs. PAV for breathing patterns, patient-ventilator interaction [83]; PSV vs. A/C for mechanoreceptor reflexes [78]; PSV vs. PAV on preserving minute ventilation [84]; PSV vs. APRV/BIPAP vs. noisy PSV for respiratory patterns [54]; Respiratory mechanics variables across different levels of pressure support [57]	PSV vs. PAV+ for sleep quality [21], [22]; PSV vs. NAVA for varying levels of assistance [23]; PSV vs. SIMV vs. APRV/BIPAP for various physiologic variables [34]; PSV vs. PAV, effects on dyssynchrony [18] PSV vs. APRV/BIPAP for oxygen cost of breathing [85];
**APRV/BIPAP** Airway Pressure Release Ventilation/Biphasic Positive Airway Pressure	APRV/BIPAP effects on oxygenation [33], [61]; APRV/BIPAP vs. CMV [42], [14]; APRV/BIPAP vs. PC-IRV [45]; APRV/BIPAP vs. PSV [50], [59], [56]; APRV/BIPAP vs. VC-IRV for oxygenation [27], [42]; APRV/BIPAP vs. PCV [12], [40], [29], [56], [53]; APRV/BIPAP vs. noisy PSV [54]	APRV/BIPAP effects on hemodynamics [33]; APRV/BIPAP effects on organ perfusion, cardiac indices [62], [28]; APRV/BIPAP vs. CMV for pericardial pressures, cardiovascular function [32]; APRV/BIPAP vs. PCV for cardiac output [61], [40], [29]; APRV/BIPAP vs. PSV [29]	APRV/BIPAP vs. SIMV [34], [11]; Respiratory dynamics [86]; APRV/BIPAP vs. PSV for work of breathing [85], [29]; Effect on respiratory muscle blood flow, respiratory work [75]; APRV/BIPAP vs. CMV [14]; APRV/BIPAP vs. PCV, A/C [29]; APRV/BIPAP vs. PCV for transpulmonary pressure [40]	APRV/BIPAP vs. VC-IRV for sedation [26]; Effects on GI tract, regional blood flow [62], [63] Effect on analgo-sedative [16], [11]
**PAV** Proportional Assist Ventilation	PAV vs. PSV [79], [80]	PAV vs. PSV [38]	PAV vs. PSV for workload relief [70], [71], [84]; Effects on respiratory system variables and work of breathing [68], [87], [77], [69]	PAV vs. PSV for dyssynchrony and sleep [21], [22], [18]; PAV vs. PSV for breathing patterns and patient-ventilator interaction [83]; PAV vs. NAVA for levels of assistance [23]; PAV vs. CPAP [47]
**NAVA** Neurally Adjusted Ventilatory Assist			Unloading of respiratory muscles [72]; Effects on diaphragmatic activity [66]	NAVA vs. PAV [23]; NAVA effects on VILI [89]

CPAP: continuous positive airway pressure; GI: gastrointestinal; IRV: inverse-ratio ventilation; P-ACV: Pressure-controlled assist ventilation; VILI: ventilator-induced lung injury.

### Study characteristics

#### Description of the RCTs

Putensen and colleagues [12] evaluated the long-term effects of gas exchange and cardiovascular function of spontaneous breathing (APRV/BIPAP) during ventilatory support in 30 polytrauma patients with injury severity scores (ISS) >40 and ALI, thus at risk of developing ARDS. After initial stabilization, the patients were randomized to either APRV/BIPAP or pressure-limited, time-cycled controlled mechanical ventilation (PCV) for 72 hours; patients in the PCV group were subsequently weaned using APRV/BIPAP. The study found that partial ventilatory support using APRV/BIPAP enabled reductions in sedative medications, contributed to improvements in arterial oxygenation, pulmonary compliance and systemic blood flow, and was associated with a decreased duration of mechanical ventilation and ICU LOS. With regard to study methods, there was no description of random sequence generation, allocation concealment, or adherence to the intention-to-treat (ITT) principle. Blinding to the intervention was not feasible, however, follow-up was complete.

Varpula and colleagues [11] compared APRV/BIPAP to SIMV-PC/PS – two partially ventilatory support approaches - in patients with early ARDS. Patients were randomized if the P/F was <200 mmHg after a 2–24 h stabilization phase with pressure A/C ventilation. Of note, both modes consisted of time-cycled, pressure-controlled mechanical breaths and differed only in the synchronization of mechanical breaths with spontaneous efforts and the support of additional breaths. Similar ICU-free days were reported for APRV/BIPAP and SIMV (11.9±1.7 versus 10.7±1.4 days, respectively) (p-value not reported). A non-significant trend toward more VFD with APRV/BIPAP (RR 1.2 (−3.4; 5.4) days) was reported. This unblinded trial used concealed allocation, however, no information about an ITT analysis was provided. The study was stopped early due to futility, so was underpowered for planned efficacy analyses.

### Primary Outcomes

Studies reporting primary outcomes (mortality, ICU and hospital LOS, duration of mechanical ventilation and/or VFDs) are summarized in Table 3; the highest level of evidence (LOE) as per the modified Oxford system (Table 1) is indicated in adjacent parentheses.

**Table 3:** Summary of results for clinical studies.

Author	Groups	Groups Matched	Mortality	ICU LOS	Ventilator Free Days	Repiratory & Haemodynmics Data Other Endpoints of Interest
(Level)		(n)	(%)	(days)	(VFDs)	
Putensen 2001 (**1b)** [12]	Trauma pts. at risk of ARDS; APRV/BIPAP vs. PCV	Yes (30)	20% APRV/BIPAP vs. 26% PCV (p = ns)	23±2d vs. 30±2d (p<0.05) APRV vs PCV	15±2d vs. 21±2d, APRV vs. PCV (p<0.05)	APRV associated with increased C_RS_, PaO_2_, CI, DO_2_ (p<0.05); decreased O_2_ extraction (p<0.05); pts with PCV needed higher doses of sufentanil, midazolam, norepi-nephrine, dobutamine (all p<0.05).
Varpula 2004 [11] (**1b)**	APRV vs SIMV+PS in adult pts with early ARDS	Yes (58)	17% APRV vs. 18% SIMV (p = 0.91)	11.9±1.7 vs. 10.7±1.4 ICU-free days APRV vs. SIMV-PS	13.4±1.7 vs. 12.2±1.5 for APRV vs. SIMV-PS	Inspiratory pressure 25.9±0.6 vs 28.6±0.7 cmH_2_0 for APRV vs SIMV-PS (p = 0.007); improved organ function: SOFA-score decreased by 2.8±0.8 vs 1.7±0.2 (APRV vs SIMV), LIS decreased 0.8±0.1 vs 0.6±0.2 (APRV vs SIMV) *stopped early for futility
Cereda 2000 (**2b)** [15]	PSV for ALI in pts. on CPPV ×24 h	No (48)	27% (none died during study period)	NR	NR	38/48 remained on PSV, 10/48 failed transition; successful application of PSV in ALI/ARDS.
Rasanen 1991 **(2b**) [14]	APRV vs. CMV in ALI	No; Crossover (50)	30%	NR	NR	Peak airway pressure lower in APRV (28±12 vs 55±17 cmH_2_O, p<0.001); no adverse effects or complications.
Varpula 2003 (**2b)** [16]	APRV/BIPAP vs. SIMV to reduce proning in ARDS	No (45)	8%(APRV/BIPAP) vs. 14% (SIMV) at 28 days	NR	NR	Before first prone episode, P/F better (P = 0.02) in APRV/BIPAP than SIMV-PC/PS (162 vs 123 mmHg); APRV enhances response to prone positioning.
Cane 1991 (**2b)** [13]	APRV (efficacy) in severe ARDS	No (14)	67%	NR	NR	V_T_ lower (0.79±0.11 vs 1.05±0.15 litres, P = 0.0002) with APRV/BIPAP vs. CPPV; P_MAX_/P_INFLATION_ lower (38.9±10.1vs 64.6±15.4 cmH_2_O, p = 0.0001) with APRV/BIPAP vs CPPV
Fan 2008 **(2b)** [17]	APRV/BIPAP vs. A/C: analgo-sedative doses, sedation status in ARDS	No (165)	12% vs. 49% (p = 0.004) APRV/BIPAP vs. A/C	14 vs. 10 days (p = 0.04) for APRV/BIPAP vs. A/C	NR	Significantly lower analgosedative doses, improved sedation status (RASS −2 for APRV vs −4 for A/C, p = 0.002), on day 1 post ALI, also observed in patients with lower APACHE2 scores (<20); differences in patient characteristics, practice may have contributed.
Xirou-chaki 2008 (**2b)** [18]	PAV+ vs. PS in mixed ARF including ARDS (64/208 patients)	Yes (208)	NR	NR	NR	11.1 vs 22% (p = 0.040) failed PAV+ vs PSV; patient-ventilator dyssynchony 5.6 vs 29% (p<0.001) for PAV+ vs PSV; proportion of patients meeting criteria for unassisted breathing did not differ between modes; PAV+ may be used in critically ill patients and increases the probability of continued spontaneous breathing.
Dart 2005 **(3b**) [19]	APRV in patients at risk of ARDS	No (46)	9%	17+/−7d	NR	Alveolar recruitment : 13% improvement in release V_T_ (p = 0.02) w APRV, 23% improvement in PaO2/FiO2 (p = 0.017) P_AWP_ decreased 19% (p = 0.001).

APACHE2: Acute Physiology and Chronic Health Evaluation 2 Score; ARF: acute renal failure; CI: cardiac index; CPPV: continuous positive pressure ventilation; CRS: compliance (respiratory system); DO2: oxygen delivery; PaO2: partial pressure of oxygen (mmHg); PMAX/PINFLATION : upper and lower (respectively) pressure levels in APRV/BIPAP mode; LIS: lung-injury score; NR: not reported; RASS: Richmond Agitation Severity Score; SOFA: sequential organ failure assessment; VT: tidal volume; all other abbreviations as stated previously in the text.

#### 1. It is unclear whether partial ventilatory support modalities improve survival of patients with ALI and ARDS

One RCT (n = 58, LOE 1b) reported a 28-day mortality of 17% and 18%, respectively, between two partial ventilatory support modes (APRV/BIPAP vs. SIMV-PS) [11] but did not include a controlled ventilation strategy group. This trial was stopped early, so was underpowered to show differences between ventilation strategies. The other RCT [12] (LOE 1b) of 30 patients compared APRV/BIPAP with PCV for 72 h and reported no difference in mortality. Six non-randomized level 2b studies and one level 3b study report mortality, however rates are variable among studies (9–67%, Table 3) [13]–[19] and do not permit conclusions due to inclusion of a single arm.

#### 2. It is unclear whether partial ventilatory support modes decrease LOS in ICU in patients with ALI/ARDS

*A s*tatistically significant decrease in ICU LOS was reported in one RCT: 23±2 days in the APRV/BIPAP group compared with 30±2 days in the PCV group (p<0.05) [12] (LOE 1b), however patients in the PCV arm received more sedation and neuromuscular blockade. It is therefore unclear whether the observed effect on LOS is attributable to the ventilation mode or the sedation regimen.

#### 3. It is unclear whether early application of partial ventilatory support modalities decreases the duration of mechanical ventilation

One RCT including 30 patients [12] demonstrated a clinically and statistically significant reduction in time on a ventilator (15±2 in APRV/BIPAP group versus 21±2 days in PCV group, p<0.05) (LOE 1b). The other RCT [11], involving 58 patients, found no difference in VFDs between APRV/BIPAP (13.4±1.7) compared to SIMV+PS (12.2±1.5, p = 0.83) (LOE 1b), however no comparison to controlled ventilation was made. An observational study of 165 ARDS patients demonstrated that APRV/BIPAP increased the duration of ventilation when compared to A/C (14 versus 10 days, p = 0.04) [16] (LOE 2b); that said, important between-group differences included a greater number of surgical and trauma patients in the APRV/BIPAP group, fewer admissions for primary respiratory disease, a lower comorbidity index and lower APACHE II scores.

### Secondary Outcomes

#### 1. PAV improves the quality of sleep in ventilated ALI patients; it is unclear if this outcome applies to other modes of partial ventilatory support equally

Two studies demonstrated improved [21] or similar [22] sleep patterns in patients ventilated with PAV as compared to controlled modes, possibly attributable to improved patient-ventilator synchrony (LOE 4b).

#### 2. Some partial ventilatory support modes alleviate patient-ventilator asynchrony more efficiently than others

In their prospective cross-over study comparing NAVA and PSV, Colombo and colleagues [23] demonstrated improved patient-ventilator synchrony with NAVA without compromising respiratory physiologic variables; synchrony was measured with the asynchrony index that incorporates wasted efforts and double triggering (LOE 4b). Four prospective clinical studies demonstrated improved patient-ventilator interaction with PAV [21], NAVA [23], Adaptive Support [24] and Volume Assured Pressure Support [25] compared to controlled modes (LOE 4b). Animal data further suggests improved diaphragmatic unloading [26] during NAVA (LOE 5a).

#### 3. The application of partial ventilatory support modalities may decrease the utilization of analgesic, sedative and neuromuscular blocking agents

Three observational studies demonstrated dramatic decreases in the doses of sedatives and analgesics and the ability to avoid the use of neuromuscular blockers [27], [28], [30] (LOE 2b, 4b); the use of neuromuscular blockers ranged from 3.4% in A/C [30] (LOE 2b) to 74% in PCV [28] (LOE 4b).

#### 4. Preserved spontaneous breathing with partial ventilatory support may have beneficial effects on gas exchange and oxygenation

Twenty three clinical studies (LOE 4) demonstrated either preserved [13]–[15], [23], [31], [33]–[39] or improved [11], [16], [19], [41]–[48], [54], [57] oxygenation using partial compared to full ventilatory support modes, which is supported by robust experimental data [29], [32], [40], [49], [50], [52], [53], [55], [56] (LOE 5). Conversely, only one study found improved oxygenation with controlled ventilation compared to PSV [51] (LOE 5).

#### 5. Partial ventilatory support modalities have the potential to improve ventilation/perfusion distribution in ALI and ARDS

Two studies reported better V/Q matching with partial ventilatory support in patients with ALI/ARDS [54], [59] (LOE 4). This finding has been consistently demonstrated in animal studies [29], [40], [51], [53], [56]–[58] (LOE 5) and is likely due to preserved contraction of the diaphragm and reduced attendant atelectasis in the dependent regions of the lungs [76]. Only one study reported abnormal breathing patterns with unsupported spontaneous breathing, resulting in smaller tidal volumes and worsened V/Q matching. Patients in this study were difficult-to-wean patients with chronic obstructive lung disease prior to development of ALI [60].

#### 6. Partial ventilatory support modalities may maintain or improve hemodynamic parameters

Several human physiologic studies (LOE 4) demonstrated that hemodynamic parameters (cardiac output, arterial blood pressure) were either maintained or slightly improved when partial ventilatory support modes were used [28], [32]–[34], [39], [42], [43], [46], [54], [59], a finding demonstrated consistently throughout the animal studies [29], [35], [61] (LOE 5).

#### 7. Partial ventilatory support modalities preserved organ function and improved blood flow to, and oxygenation of, several cardinal organs

Function of specific organ systems was seldom a study endpoint, however, using lactate as a surrogate marker of tissue perfusion Kaplan and colleagues [28] (LOE 4b) demonstrated improvements in lactate and urine output with APRV/BIPAP as compared to PCV. Improved renal perfusion and function has been demonstrated in humans [62] (LOE 4b); improvements in intestinal [63] and portal blood flow [64] have been demonstrated in pre-clinical studies (LOE 5).

#### 8. Spontaneous breathing with partial ventilatory support modes preserves diaphragmatic function

Controlled ventilation without patient effort is linked to ventilator induced diaphragmatic dysfunction within 18 hours after initiation of mechanical ventilation [65]. Experimental studies with use of intramuscular electrodes inserted into the diaphragm [67] have demonstrated improved diaphragmatic function and attenuation of diaphragmatic atrophy with partial ventilatory support modes [26], [66] (LOE 4, 5).

#### 9. The effects of partial ventilatory support modalities on ventilation associated lung injury are undetermined

No studies specifically evaluated the effects of preserved spontaneous breathing on ventilator associated lung injury.

#### 10. Partial ventilatory support modalities demonstrate variable, but non-detrimental, effects on work of breathing; there are differences between modes, and modes using volume regulation are associated with increased work of breathing when compared to those using pressure regulation

The majority of studies assessing the workload and oxygen cost of breathing compared a controlled mode to PAV, which provides load-adjustable gain factors for volume and flow assist. These studies consistently demonstrated work of breathing was not increased compared with controlled modes, and could even be offloaded to near normal with PAV [47], [68], [69], [71] (LOE 4b and 6) and NAVA [72] (LOE 4b). Among partial support modes, SIMV (with volume control) increased the work of breathing in ALI patients as compared to A/C [43] and PS [73], both of which allowed progressive muscle unloading (LOE 4b). The work of breathing was also higher using BIPAP/APRV compared with A/C or PS [29], [74] (LOE 5,6). The authors of two different studies noted significantly decreased or stable workloads with use of pressure support modes [25], [38] (LOE 4b). One experimental study [75] demonstrated increased respiratory work and respiratory muscle blood flow in ALI during spontaneous breathing at ambient airway pressure. The institution of APRV mitigated these effects (LOE 5).

## Discussion

We reviewed studies that investigated partial support modes of mechanical ventilation in patients with ALI and ARDS; we found that the reported beneficial effects of preserved spontaneous breathing were mainly physiological effects, demonstrated as improvement of respiratory function and hemodynamic stability. Only two RCTs have been conducted, and only one of these compared partial support with controlled ventilation. One demonstrated a shorter ICU length of stay, and the other more ventilator-free days, suggesting that further research is required to understand the impact of partial support strategies on clinical outcomes. Unfortunately, the heterogeneity of published investigations generated more questions than they have answered, from both methodological and clinical points of view.

Based on this review, we can neither conclude nor refute the statement that the use of partial ventilatory support modalities in patients with ALI and ARDS impacts survival or duration of mechanical ventilation and lengths of ICU stay. However, we can conclude that partial ventilatory support modes can be successfully utilized in the early phases of ALI.

### Important physiologic effects

One of the major contributing factors to hypoxemia in ALI and ARDS is dependent lung atelectasis and accompanying V/Q mismatch that should –because of preserved diaphragmatic contraction- improve during spontaneous breathing. Most studies did in fact demonstrate the superiority of partial ventilatory support modes over controlled modes in the restoration of gas exchange, benefits that were observed without an increase in the oxygen cost of breathing. Other advantages were improved cardiac outputs and indices, overall hemodynamic stability (blood pressure), and organ blood flow [63], [75]. The supporting evidence is stronger in pre-clinical studies, and is consistent across different species and models of experimental ALI (e.g., hydrochloric acid [HCl]- aspiration, oleic acid- or lavage induced).

Some partial ventilatory support modes, especially PAV or NAVA, may improve patient-ventilator synchrony better than others. However, while improved sleep quality has been demonstrated in clinical studies, no impact on mortality or duration of ventilation has been shown.

Theoretically, the reduction in (inspiratory) airway pressures associated with partial ventilatory support holds the potential to attenuate VALI, although no studies addressing this outcome were identified within the search period. Recent experimental studies have examined VALI in association with preserved spontaneous breathing, with promising results [88], [93].

### The use of neuromuscular blocking agents in patients with ARDS

Given that more than 30% of patients received chemically paralysis when recruited into an ARDS trial [1]–[3], physicians may be less likely to use partial ventilatory support modes in the acute phase of ARDS. In the past, the absence of highly adaptive, fast-response ventilation modes made it reasonable to “adjust the patient to the ventilator” for prevention of patient-ventilator dyssynchrony and desaturation. A recently published trial highlights this approach. Patients with severe ARDS (P/F≤150 mmHg) were randomized to receive cisatracurium or placebo for 48 h, and the adjusted 90-day survival and VFDs improved without increased muscle weakness in the paralysis group [94]. Both study groups were ventilated using a volume-regulated, controlled mode that typically requires heavy sedation. Of note, in *both* arms the patients' breathing was equally abolished in order to “rest the lung”. Both arms also permitted the administration of additional open label cisatracurium to control patient-ventilator dyssynchrony; this occurred in ≥50% of patients in either arm. Although an improvement in clinically important outcomes was achieved with neuromuscular blockade, it is important to note that the study did not compare partial ventilatory support versus controlled ventilation. Therefore, while certain conclusions about the potential benefits of using neuromuscular blockade in ALI/ARDS patients ventilated using volume-controlled modes can be drawn, such conclusions are not generalizable to the ARDS population in general. For patients with severe ARDS who do require very invasive ventilator settings (e.g., those requiring very high PEEP and FiO_2_ levels, or exhibiting severe dyssynchrony with the ventilator), partial ventilatory support modes may not be appropriate. Another approach to this patient population might even be the use of extracorporeal techniques, as evaluated in two recent trials investigating the potential to improve survival in patients with severe ARDS with the use of extracorporeal membrane oxygenation and/or carbon dioxide removal [95], [96]. Although neither study explicitly evaluated partial ventilatory support, extracorporeal gas exchange has the potential to unload the patients' lungs and thus to reduce the minute ventilation that needs to be provided by mechanical assist.

### Grading of studies and interpretation of results

Using a modified version of the Oxford system might invite criticism as not representing consensus. The difficulty of combining low-grade clinical evidence with a wealth of experimental studies has prompted us to modify the existing system. It also factors in the finding that most clinical studies were of very short duration and targeted physiological parameters only (i.e. oxygenation) that have no correlation with the outcomes of interest. For the same reason we have abstained from making recommendations based on the level of evidence, such as with the GRADE system [90].

### Mode definition and terminology

A major difficulty in undertaking this review was the lack of consistency throughout the literature when it came to naming and defining the myriad different modes of ventilation used. As examples, A/C can be applied either as a controlled or as an assisted mode; and, without spontaneous breathing efforts, A/C becomes equivalent to volume-controlled ventilation, and APRV/BIPAP becomes equivalent to PCV. Thus, the differences between APRV, BIPAP or PC-SIMV lie in the details of the ventilation settings utilized, the nuances of which are often underappreciated by clinicians, authors and readers alike. In addition, the way that mechanical breaths are administered during partial ventilatory support, differ: they may be time-cycled and triggered (A/C, SIMV, APRV/BIPAP) or unsynchronized (BIPAP), flow-cycled (PSV, PAV) or “other” (NAVA). The patient may be able to increase the number of supported breaths (A/C, PSV, PAV, NAVA) or take additional unsupported breaths (SIMV, APRV, BIPAP), or breaths buttressed with pressure support (SIMV-PS, BIPAP-PS). Depending on the strategy used, the effects on alveolar recruitment, V/Q mismatch and work of breathing may be significant for a given mode [29], [50], [51], [58], [59], [61]. A similar problem is encountered with the mode settings, as they also greatly influence effect. Without consistency and explicitly defining what is meant by a mode, therefore, the literature becomes difficult to interpret.

So, for the purpose of this review we evaluated and grouped modes according to their intended use by their respective authors, but this does not preclude the possibility that effects of different modes might have been lost due to grouping. With the exception of PSV, the patients' contribution to the work of breathing may be decreased to nothing. Preliminary reports of patients with ALI/ARDS have found spontaneous breathing efforts are present in only 20% of patients ventilated with A/C [91]. Additionally, since the only RCTs to date focused solely on APRV/BIPAP, it remains undetermined whether there is an ‘ideal’ mode to use in ALI/ARDS [92].

These challenges in ventilation research are reflected by the difficulty in distinguishing between the inherent properties of specific ventilators and application of modes.

### Possible clinical implications

The beneficial physiological effects of preserved spontaneous breathing may encourage clinicians to use partial ventilatory support modalities when treating patients with ALI and ARDS, but the evidence to link its utilization with measurable improvements in clinically important outcomes remains weak. We were unable to characterize the optimal subpopulation of ALI/ARDS patients, clinical indications, or time of initiation of spontaneous modes in mechanically ventilated patients with ALI or ARDS; nor could we define the optimal amount of partial ventilatory support.

Current standard of practice [97] recommends that a daily assessment (i.e., daily spontaneous awakening and concomitant spontaneous breathing trials) be undertaken in order to identify the earliest time at which patients will tolerate a transition to partial support ventilation. With the continued evolution of partial support modes, other concepts – such as a less abrupt and more gradual increase in the patient's work of breathing as tolerated from the very early phases of illness – might become part of the standard treatment for patients with ALI and ARDS. This approach has been favored by some clinicians [5], although the benefit has yet to be proven.

### Limitations

The lack of human studies has prompted us to consider animal data as supporting evidence, a strategy that has been previously employed in other systematic reviews [98]. Although this scoping review used systematic methods (defined search and evaluation strategies, independent reviewers), the absence of clinical data with a high level of evidence weakens the strength of clinically important inferences. A similar problem exists regarding the ventilatory modes we have discussed. While it may seem an oversimplification to evaluate partial ventilatory support modes as a whole, without accounting for the potentially significant differences between modes, the evidence is not stronger if only a single mode is regarded. We have therefore discussed the differences between partial support modes from a physiologic perspective, permitting us to include experimental studies.

Based on this review, we can neither conclude nor discount that the use of partial ventilatory support modalities affect survival or reduce the duration of mechanical ventilation or lengths of ICU stay in patients with ALI and ARDS. However, partial ventilatory support modalities can be effective in moderate to less severe ALI and ARDS, and have the potential to decrease the duration of mechanical ventilation.

The impact on survival and ICU LOS remain, as of yet, undetermined. Physiologically, partial ventilatory support modalities reduce the need for sedation and neuromuscular blockade; they improve respiratory system mechanical function and contribute to better gas exchange and hemodynamic function. In addition, improved patient-ventilator synchrony, comfort, and sleep patterns have been demonstrated.

We were not able to characterize an optimal subpopulation of ALI/ARDS patients, clinical indications, or a time point for initiation of spontaneous modes in mechanically ventilated patients with ALI or ARDS; nor could we define the optimal amount of partial ventilatory support. Further research is required to answer these questions.

## Supporting Information

File S1 **Outline of search strategy.**
(DOC)
