# Supplementary material for: Partial Ventilatory Support Modalities in Acute Lung Injury and Acute Respiratory Distress Syndrome—A Systematic Review
Source: PLoS One. 2012 Aug 16;7(8):e40190. doi: 10.1371/journal.pone.0040190 (PMC3420868; doi:10.1371/journal.pone.0040190)
Supplement: File S1 — Outline of search strategy. (DOC) [file pone.0040190.s001.doc]

**Partial Ventilatory Support Modalities in Acute Lung Injury and Acute Respiratory Distress Syndrome – A systematic review.**

Sarah M. McMullen, MD, MSc^1^, Maureen Meade, MD MSc^2^, Louise Rose, RN MN PhD^3^, Karen Burns, MD MSc^4^, Sangeeta Mehta, MD^5^, Robert Doyle, MD^1^, Dietrich Henzler, MD PhD^1^for the Canadian Critical Care Trials Group (CCCTG)

Electronic Supplementary Material

Search strategy

The search was performed in MEDLINE (1966-February 2009), Cochrane, and EmBase (1980 - February 2009) databases for articles published in English, French or German (languages accessible to the investigators)

1. “neurally adjusted ventilatory assist”
2. NAVA
3. “proportional assist ventilation”
4. PAV
5. IPPV
6. “inspiratory positive pressure ventilation”
7. “biphasic intermittent positive airway pressure”
8. “assisted ventilatory support”
9. “spontaneous breathing”
10. “synchronized intermittent mandatory ventilation” or SIMV
11. “partial ventilatory”
12. “partial ventilation”
13. “bilevel ventilation”
14. CPAP
15. “Biphasic continuous positive airway pressure”
16. “bilevel continuous positive airway pressure”
17. “airway pressure release ventilation” or APRV
18. "Positive-Pressure Respiration"[Mesh]
19. 1/19 OR
20. “critically ill”
21. "respiratory drive"
22. “respiratory depression”
23. “respiratory failure”
24. "Lung Injury"[Mesh] OR "Lung Diseases"[Mesh]
25. “respiratory distress"
26. ARDS
27. "Intensive Care Units"[Mesh]
28. "Respiratory Insufficiency"[Mesh]
29. "Respiratory Distress Syndrome, Adult"[Mesh]
30. 20/29 OR
31. "Sleep Apnea Syndromes"[Mesh]
32. “sleep apnea”
33. Noninvasive [ti]
34. 31/33 OR
35. #19 AND #30
36. #35 NOT #34
37. Limit to All Child
38. #36 NOT #37 = 6328
39. Limit: English, French & German = 5652
